# Educational Requirements

**Published:** 1915-09

**Authors:** 


### Educational Requirements.

This is addressed, by request to physicians having sons, other relatives, or young men in whom they are personally interested. Do not encourage such young men to enter medicine unless they have a genuine preference for that calling and at least a moderate degree of fitness for the work, including patience, tact and good health and habits. Give them, also, a fair understanding of the favorable and unfavorable factors, economic and otherwise, incident to medical practice.

Granted that the decision in favor of a medical career has been made, insist that the young man shall fulfill the requirements in a liberal spirit, not in that of getting the medical license with a minimum of time, education and work. It is easy to read the signs of the times. By the time any youth who is now about to start on his medical training is really engaged in active practice, most states will have required two years of college work as a basis for commencing the medical course and, probably, several of the leading institutions will require a full college training, with bachelor's degree.

There are, in the main, four objections to the elevation of strictly educational requirements for medical license or any other similar privileges: 1. The poor boy argument; 2. The argument that what was good enough for one generation is good enough for the next; 3. The sacrifice of time, especially in consideration of the rather small emoluments of medical practice; 4. The lack of practicality of education along scholastic lines.

The first one does not impress us very forcibly. There is no form of philanthropic assistance more freely rendered and more widely available and none which may be received with such entire fredom from loss of self-respect, than free tuition, help to self-support or favors toward current expenses while getting an education. It is no favor to the poor boy to give him a poor training and no genuine indication of democratic tendencies to emphasize the opportunity for advancement without insistence on a corresponding improvement of service.

In very few instances can the standards of one generation be properly applied to the next. The college represents no greater hardship nor effort today than did the rudimentary training of the little red school house, two generations ago, nor that of the academy or high school, a generation ago. For some reason or other, the real scholarship, mental ability and progressive education of the individual seems to depend rather on the proportionate effort made toward formal schooling than on the actual amount received and, at present, there is not the same expectation of mental advance beyond school days as there was formerly. Thus, the man who resents the higher education of his son as a reflection on his own attainments, fails to grasp the real distinction between formal and informal, post-graduate education.

The sacrifice of time involved in a general advance of requirements is a very serious problem. The late William Pepper expressed the opinion that any system of educational requirements should allow the youth to enter upon his life work, in medicine, by the time he was twenty-three. However, with greater attention to individual mental ability and the possibility of more accurate grading in dealing with more concentrated population, it is possible to shorten the grammar school period for many children and, with more mature and more uniformly better trained medical students, the medical course itself can be, if not shortened, at least held to its present time. As higher educational requirements tend automatically to restore the balance between supply and demand and as the number of medical positions supported by some branch of the government, it will also be possible to compensate by a shorter period of non-employment, for the greater time spent before receiving the license.

While it is the custom to regard higher education as impractical, actual experience disproves this idea. From a narrow financial standpoint, the young man contemplating a life-work—or his older advisor for him—should take into consideration the increasing number of special positions for which a college education is either formally required or counted as a favorable item in making selections. One of the largest electric companies of the country regularly picks over the graduating classes of two or three technical schools yearly not so much for its strictly scientific purposes as for the rank and file of its executive and commercial positions. Modern business generally depends largely on college graduates and even in politics, we note the same tendency. So far as the professions are concerned, it may almost be said that there is a popular demand for a college education, though we due qualifications.

If one reviews the progress of medicine for the last few years, it can scarcely be questioned that it has been due almost entirely to the broader interpretation of technical facts by knowledge that would be superficially regarded as impractical and, certainly, as pre-medical, so far as arrangements of courses are concerned. Almost all the elaborate instruments and apparatus by means of which medicine has made its astonishing progress, depend upon knowledge which the average person must gain between the high school and the medical college. Even in legislation to advance matriculation standards, we note the influence of ''practical'' rating of subjects. Chemistry, physica, allied sciences and modern languages are recognized as ''practical'' for the medical student although, unfortunately, the amount required in each of these subjects is scarcely adequate to fulfill the hope of advanced research based on their principles. In reviewing books of many fields in medicine, we have been frequently surprised at the inclusion of higher mathematics and at hints of the remarkable developments that a more general familiarity with these branches, and with practical medicine, might bring about. In fact, the more one observes, the more confused he becomes in attempting to distinguish between useless educational lumber and information, apparently of the most ''scholarly'' and unutilitarian kind, which affords a basis for the most practical results.

In conclusion, we wish to emphasize the fact that Personal, in the title of this article, does not apply to the editor but to the reader who is somehow responsible for the immediate future of a prospective practitioner of medicine.

